# Exploring the Therapeutic Potential of MIR‐140‐3p in Osteoarthritis: Targeting CILP and Ferroptosis for Novel Treatment Strategies

**DOI:** 10.1111/cpr.70018

**Published:** 2025-03-05

**Authors:** Feng Ma, Lexin Wang, Hao Chi, Xinyi Li, Yaoqin Xu, Kexin Chen, Jingfan Zhou, Runqin Yang, Jie Liu, Ke Xu, Xiaoling Yang

**Affiliations:** ^1^ NHC Key Laboratory of Metabolic Cardiovascular Diseases Research Ningxia Medical University Yinchuan China; ^2^ General Hospital of Ningxia Medical University Yinchuan China; ^3^ Western Institute of Digital‐Intelligent Medicine Chongqing China; ^4^ Clinical Medical College, Southwest Medical University Luzhou China; ^5^ Ningxia Key Laboratory of Vascular Injury and Repair Research Yinchuan China; ^6^ Department of General Surgery Dazhou Central Hospital Dazhou China; ^7^ Department of Oncology, Chongqing General Hospital, Chongqing University Chongqing China

## Abstract

Osteoarthritis (OA) is a prevalent and debilitating joint disorder that affects millions of individuals worldwide, severely impairing mobility, independence, and quality of life. Emerging evidence suggests that ferroptosis is a critical factor in OA pathogenesis. However, its precise involvement and underlying mechanisms remain poorly understood. In this study, we first identified that cartilage intermediate layer protein (CILP) mediates the regulation of ferroptosis‐related genes in OA through hdWGCNA analysis combined with single‐cell RNA sequencing. Further investigation revealed a significant upregulation of CILP protein expression in C28/I2 cells under LPS induction. Mechanistically, bioinformatics analysis identified differentially expressed miRNAs; qRT‐PCR combined with a dual‐luciferase experiment revealed that miR‐140‐3p was downregulated and directly targets CILP. Experimental data further demonstrated that miR‐140‐3p regulates ferroptosis, inflammation, and oxidative stress by targeting CILP. These findings offer valuable insights into the molecular mechanisms of the miR‐140‐3p/CILP axis in regulating ferroptosis, inflammation, and oxidative stress, thus providing a foundation for developing therapeutic strategies for OA.

## Graphical abstract

Global landscape.

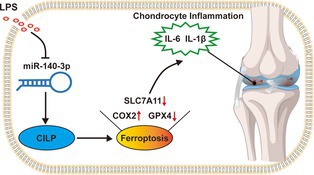

## 1 Introduction

Osteoarthritis (OA) is a prevalent and debilitating condition that causes pain, reduces quality of life and imposes significant healthcare costs [1, 2]. Despite the availability of current treatments, OA remains highly prevalent, particularly among individuals over 50 years of age [3]. Ferroptosis, a form of regulated cell death distinct from apoptosis and necrosis, is primarily driven by oxidative stress (OS), which induces lipid peroxidation and damages cell membrane structure and function [4, 5]. Under abnormal iron metabolism, excess or misallocated iron exacerbates OS, promoting ferroptosis [6]. Several studies have confirmed the involvement of chondrocyte ferroptosis in OA progression. However, the pathogenesis and potential therapeutic interventions targeting ferroptosis in OA are not yet fully understood.

Recent research has highlighted the crucial roles of non‐coding RNAs, particularly microRNAs (miRNAs), in the regulation of complex diseases [7, 8]. MiRNAs, which are noncoding single‐stranded RNAs approximately 20–24 nucleotides in length, regulate gene expression by binding to the 3′‐untranslated region (3′‐UTR) of target messenger RNAs (mRNAs), leading to their degradation or inhibition of translation [9]. Altered expression of miRNAs has been linked to the development of OA, and interestingly, certain miRNAs have been identified as regulators of chondrocyte ferroptosis [10]. For example, Zhou et al. [11] showed that miR‐1 can inhibit chondrocyte ferroptosis and slow OA progression by targeting CX43. Similarly, Wang et al. [12] demonstrated that miR‐181b could prevent ferroptosis by targeting the ferroptosis‐protective protein SLC7A11, with downregulation of SLC7A11 reversing the inhibition of chondrocyte ferroptosis by antagomiR‐181b and promoting OA progression. These findings highlight the significance of investigating miRNAs in OA and their role in modulating chondrocyte ferroptosis.

The combination of high‐throughput sequencing (HTS) and bioinformatics analysis has become a valuable research tool in modern medicine, providing crucial insights for disease diagnosis, prognosis and treatment. Recent advances in bioinformatics and molecular biology have enhanced our understanding of how abnormalities in iron metabolism influence ferroptosis at the cellular level, contributing to the development and clinical features of bone and joint diseases [13]. For instance, by analysing gene expression changes and signalling pathways, researchers have begun to reveal the potential role of ferroptosis in these conditions, offering new scientific insights for developing targeted therapeutic strategies [14]. This study conducted bioinformatics analysis on data from OA cases and normal subjects to screen for differentially expressed genes (DEGs). Among these, cartilage intermediate layer protein (CILP) was identified as a key regulator in the onset of OA. CILP, a matrix protein located in the middle of human articular cartilage, is implicated in several cartilage‐related diseases [15]. Additionally, OA‐associated miRNAs were obtained from the Gene Expression Omnibus (GEO) database. Through experimental verification and bioinformatics analysis, miR‐140‐3p was identified as a potential target molecule closely linked to OA development.

MiR‐140‐3p, a miRNA derived from pre‐miR‐140, has been implicated in various diseases by regulating cell proliferation, invasion, migration and inflammation [16]. Interestingly, studies have found that miR‐140‐3p is differentially expressed in chondrocytes and is involved in OA progression. Cheng et al. [17] found that the knockdown of circ_0136474 alleviated IL‐1β‐induced damage in CHON‐001 cells through modulation of the miR‐140‐3p/MECP2 axis, suggesting a novel therapeutic target for OA. Hu et al. [18] reported that the overexpression of miR‐140‐3p in BMSC‐Exo protected joints and delayed OA pathogenesis. Furthermore, miR‐140‐3p has been identified as a potential biomarker in the molecular mechanisms of inflammation and ferroptosis in ischemic stroke [19]. However, the mechanism by which miR‐140‐3p regulates ferroptosis in OA progression remains unclear.

In this study, we analysed GEO datasets with bioinformatics methods (e.g., PPI, WGCNA) and screened a key gene (CILP) and a miRNA (miR‐140‐3p). The study integrates cellular experiments to investigate how miR‐140‐3p regulates ferroptosis and contributes to OA progression through direct or indirect interactions with CILP. Our findings enhance the understanding of miR‐140‐3p's biological functions in OA and provide a solid foundation for the development of novel therapeutic strategies.

## 2 Materials and Methods

### 2.1 TCGA Data Acquisition and Processing

We queried the GEO single‐cell sequencing database and identified the dataset GSE169454, including three normal and four OA samples for analysis. We created a Seurat object based on the single‐cell gene expression matrix from the selected dataset using the Seurat package in R. The Seurat package was then employed to normalise the scRNA‐seq data of each cell by applying the variable feature discovery method. Subsequently, the ScaleData and PCA functions were utilised to determine the number of principal components. After dimensionality reduction with t‐SNE, key cell types and subtypes were annotated and visualised through the Idents and DimPlot functions.

### 2.2 Functional Enrichment Analysis

Functional enrichment analysis was carried out utilising Metascape (http://metascape.org/gp) to identify pathways from the Kyoto Encyclopedia of Genes and Genomes (KEGG). The landscape enrichment map function was utilised to cluster the pathways for further analysis.

### 2.3 Non‐Negative Matrix Factorization (NMF) Analysis

The NMF method was applied to identify different subtypes of OA patients. First, potential prognostic DEGs were identified utilising univariate Cox regression analysis with the survival package in R. Sample clustering was performed utilising the “brunet” method within the NMF package. The patients were assigned to distinct subtypes based on the optimal number of clusters. Consensus heatmaps were generated to visualise the distribution patterns among the identified subtypes.

### 2.4 Cell Culture

Human normal chondrocytes C28/I2 (Otwo Biotech [Shen Zhen] Inc) were cultured with DMEM medium (Gibco, USA) enriched with 10% fetal bovine serum and 1% penicillin–streptomycin (Sigma, USA), maintained at 37°C with 5% CO_2_ in a constant temperature incubator (Thermo Fisher Scientific, USA) [20, 21, 22, 23, 24, 25, 26, 27, 28, 29]. The cells were seeded into six‐well plates and grown until they reached 80% confluence. They were then divided into two treatment groups: the control group, which was maintained under standard conditions without any treatment, and the lipopolysaccharide (LPS) group, which was exposed to 150 μg/mL LPS for 48 h. Each experimental group was performed in triplicate to ensure data consistency and reliability.

### 2.5 Cell Transfection

Short heparin RNA targeting CILP (sh‐CILP‐1, 2 and 3), scrambled short heparin RNA (sh‐NC), and miR‐140‐3p mimic and inhibitor were purchased from Gene‐Pharma (Shanghai, China). C28/I2 cells were transfected with sh‐NC, sh‐CILP, miR‐140‐3p mimic and inhibitor utilizing Lipofectamine 2000 (Invitrogen, USA). A fresh medium was added 8 h after transfection, and cells were harvested 48 h post‐transfection for further analysis. The efficiency of gene knockdown was confirmed by quantitative reverse transcription polymerase chain reaction (qRT‐PCR). Cell morphology and growth were monitored and documented under an inverted microscope. The sequences of these shRNA are shown in Table 1.

**TABLE 1:** Sequences of shRNAs used in this study.

Name	Primer sequence (5′ → 3′)
sh‐CILP‐1	5′‐GUGCCGUGUUCCAUGAAAUTT‐3′
sh‐CILP‐1	5′‐GUGCCGUGUUCCAUGAAAUTT‐3′
sh‐CILP‐1	5′‐GGAGGAAGGUGAUUUCAAATT‐3′
sh‐NC	5′‐GUGGAGUCUUCUCCUAAAUTT‐3′

### 2.6 Western Blot

Total protein was extracted from C28/I2 cells using the Total Protein Extraction Kit from Nanjing KeyGen Biotech Co. Ltd. Subsequently, protein concentration was determined with a BCA protein assay kit from the same supplier. The proteins were separated by sodium dodecyl sulfate–polyacrylamide gel electrophoresis (SDS‐PAGE) and transferred onto a 0.45 μm polyvinylidene fluoride (PVDF) membrane (Millipore, Sigma, USA). After blocking the membrane with 5% skim milk for 2 h, it was incubated overnight at 4°C with primary antibodies: CILP (1:1000; Abcam), COX2 (1:1000; Abcam), GPX4 (1:1000; Abcam), SLC7A11 (1:1000; Abcam), and β‐actin (1:1000; ZSGB‐Bio). The next day, the membrane was washed thrice with TBST for 10 min each, then incubated with HRP‐conjugated secondary antibodies (1:5000; ZSGB‐Bio) for 2 h. Protein bands were visualised and analysed utilising Image Lab image analysis software (Bio‐Rad, USA).

### 2.7 Immunofluorescence Staining

C28/I2 cells were washed with PBS and fixed with 4% formaldehyde for 30 min. After fixation, cells were permeabilised with 0.2% Triton X‐100 for 10 min and blocked with 1% bovine serum albumin (Sigma, USA) for 1 h at room temperature. CILP antibodies were diluted in an appropriate buffer and incubated with the samples at 4°C overnight to allow binding to the target protein. Following incubation, the cells were washed with PBS and then incubated with a fluorescein isothiocyanate (FITC)‐conjugated secondary antibody (Abcam) for 1 h at 37°C. Following another PBS wash, the cells were counterstained with 4,6‐diamidino‐2‐phenylindole (DAPI) for 5 min at room temperature to stain the nuclei. The expression of CILP was visualised under a confocal microscope (LSM 780, Zeiss, Germany), and the intensity and distribution of fluorescence signals were recorded.

### 2.8 qRT‐PCR

Total RNA was isolated from C28/I2 cells utilising Trizol reagent (Invitrogen, USA). The concentration and quality of RNA were assessed utilising NanoDrop 2000. RNA was reverse transcribed into cDNA utilising a reverse transcription kit (TaKaRa, Japan). For mRNA quantification, primers were designed by Sangon Biotech (Shanghai, China). For miRNA analysis, specific qRT‐PCR primers for miR‐140‐3p were designed by Bulge‐loop, which included a reverse transcription primer and a pair of qPCR primers (RiboBio, Guangzhou, China). The PCR conditions were set as follows: an initial denaturation at 95°C for 2 min, followed by 45 cycles of denaturation at 95°C for 10 s, annealing at 60°C for 5 s and extension at 72°C for 30 s. Expression levels were calculated utilising the $2^{-\Delta \Delta C_{t}}$ method, with GAPDH or U6 as an internal control gene to normalise transcript levels and account for differences in cDNA amounts. All sequences are provided in Table 2.

**TABLE 2:** Sequences of primers used for qRT‐PCR analysis.

CILP	Forward primer: TCCCAAGGCATCTAAGCGTC
	Reverse primer: AGCATCGTCTGTCTCCCTGA
HS‐GAPDH(138)	Forward primer: CAGGAGGCATTGCTGATGAT 20
	Reverse primer: GAAGGCTGGGGCTCATTT 18
U6	Forward primer:ATATATGGACGCTTCAATT
	Reverse primer:AACGCTTCGAATGCTTGT
hsa‐miR‐26a‐5p	Forward primer: GCGGCTTCAAGTAATCCAGGA
	Reverse primer: ACTGCAGGGTCCGAGGTATT
hsa‐miR‐26b‐5p	Forward primer: CGCGGCTTCAAGTAATTCAGG
	Reverse primer: ACTGCAGGGTCCGAGGTATT
hsa‐miR‐140‐3p	Forward primer: GCGGCTACCACAGGGTAGAA
	Reverse primer: ACTGCAGGGTCCGAGGTATT
hsa‐miR‐181a‐5p	Forward primer: GCAACATTCAACGCTGTCG
	Reverse primer: ACTGCAGGGTCCGAGGTATT
hsa‐miR‐338‐5p	Forward primer: CGGCAACAATATCCTGGTGC
	Reverse primer: ACTGCAGGGTCCGAGGTATT
hsa‐miR‐645	Forward primer: GGCGGCTCTAGGCTGGTAC
	Reverse primer: ACTGCAGGGTCCGAGGTATT
hsa‐miR‐1307‐5p	Forward primer:CTCGACCGGACCTCGA
	Reverse primer: ACTGCAGGGTCCGAGGTATT
hsa‐miR‐2355‐3p	Forward primer: CGGCATTGTCCTTGCTGTTT
	Reverse primer: ACTGCAGGGTCCGAGGTATT
hsa‐miR‐4454	Forward primer: GGCGGATCCGAGTCACG
	Reverse primer: ACTGCAGGGTCCGAGGTATT
hsa‐miR‐4456	Forward primer: GCGGCCCTGGTGGCTT
	Reverse primer: ACTGCAGGGTCCGAGGTATT

### 2.9 Dual‐Luciferase Reporter Assay

CILP was identified as a potential target of miR‐140‐3p, and the binding sites of miR‐140‐3p within the CILP 3′‐UTR were predicted by a bioinformatics prediction program. The 3′‐UTR sequences of both wild type and mutant CILP were cloned into the pmirGLO vector (Promega, USA) to generate the CILP‐WT and CILP‐MUT plasmids. These plasmids, along with miR‐140‐3p, mimic or mimic NC, were transfected into C28/I2 cells using Lipofectamine 2000 (Invitrogen, USA). The firefly and renilla luciferase activities were assessed after 48 h utilising a Dual‐Luciferase Reporter Assay System (Promega, USA). Relative luciferase activity was normalised to Renilla luciferase activity. All experiments were carried out in triplicate.

### 2.10 Enzyme‐Linked Immunosorbent Assay (ELISA)

Supernatant samples were collected from cultured C28/I2 cells. Commercial ELISA kits (eBioscience, USA) for IL‐6, IL‐1β, superoxide dismutase (SOD), and malondialdehyde (MDA) were used. Briefly, the supernatant was obtained by centrifuging the cell culture at 2000*g* for 10 min at 4°C. The collected supernatant was then incubated with biotin‐labeled antibodies for 1 h at room temperature, followed by incubation with an avidin‐peroxidase complex for 20 min. Afterward, TMB chromogenic solution was added for 30 min, and the reaction was terminated by adding the stop solution provided in the kit. Absorbance at 450 nm was monitored utilising a microplate reader (Molecular Devices LLC).

### 2.11 Statistical Analysis

All data and statistical analyses were processed utilising R software version 3.6.1 and GraphPad Prism 8.0. Data are expressed as the Mean ± SD from at least three independent experiments. Differences in gene expression levels between the two groups were assessed utilising the Student's *t*‐test. For comparisons across multiple groups, one‐way ANOVA was used. A *p* value of less than 0.05 was considered statistically significant.

## 3 Result

### 3.1 Unveiling OA Microenvironment by Single‐Cell RNA Sequencing Analysis

**FIGURE 1:**
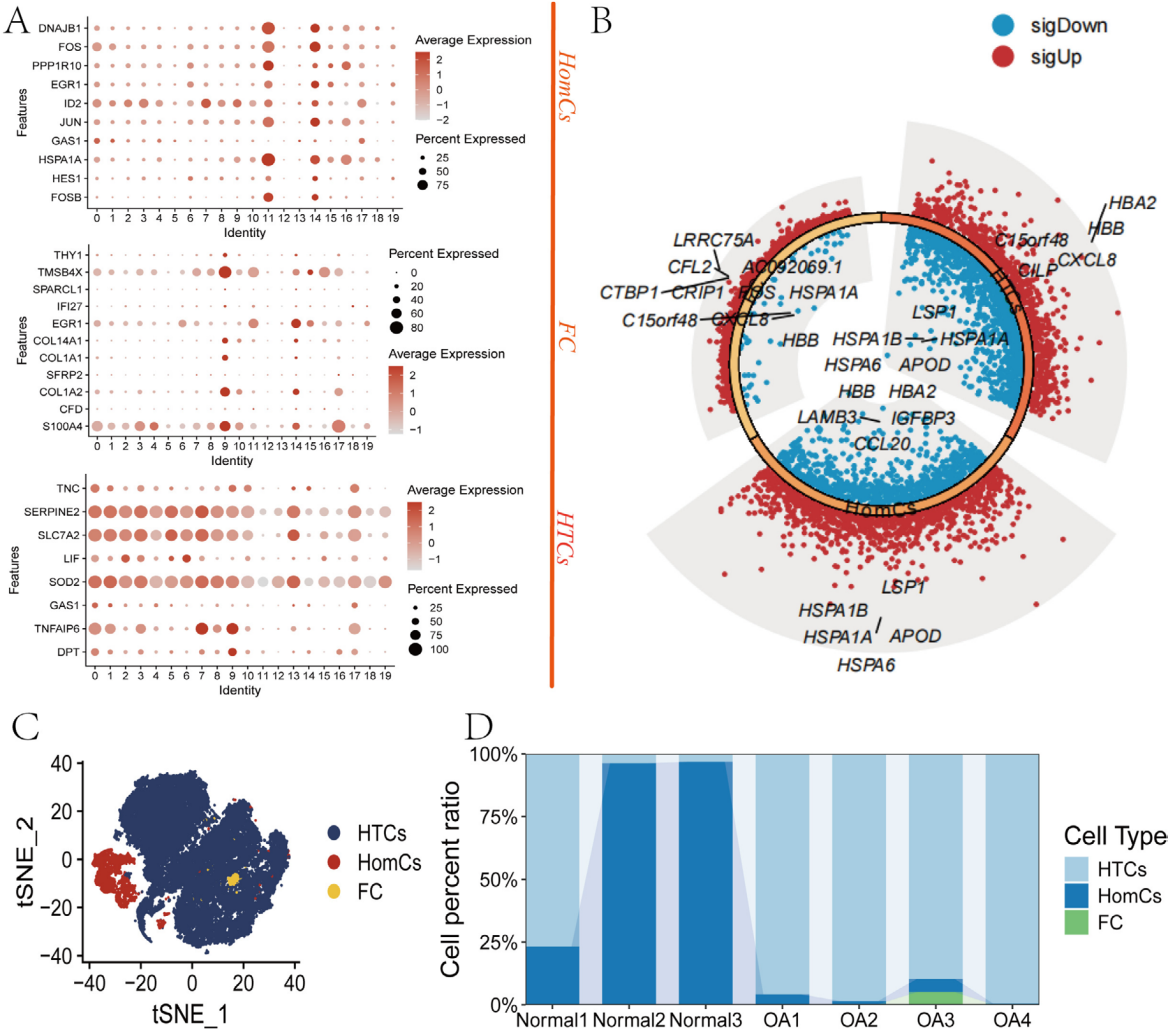
CILP expression was upregulated in OA HTC cells. (A) Annotation of single‐cell subpopulations. (B) Differential gene analysis of major cell subpopulations, with red indicating upregulated genes and blue indicating downregulated genes. (C) Visualisation of major cell subpopulations. (D) The bar chart showing the proportion classification of major cell subpopulations.

We initially selected six single‐cell datasets from OA disease models in the GEO database (three normal and four OA samples) and manually annotated the data. Three primary cell types were analysed: HTC, FC, and HomC (Figure 1A,C). Differential gene expression analysis at the single‐cell level revealed distinct expression differences among these subpopulations. CILP exhibited marked differential expression in the HTC group (red indicating upregulation, blue indicating downregulation; selection criteria: Log FC > 1 and *p* < 0.05). Notably, differences in the proportions of these cell subpopulations were observed between OA and normal samples, with CILP expression particularly elevated in HTC cells (Figure 1B). Additionally, the proportions of all cell types in normal and OA samples showed a similar distribution (Figure 1D). These findings suggest that abnormal expression in HTC cells may play a key role in exacerbating OA‐related damage.

### 3.2 Identification of CILP in HTC‐Specific Hub Genes by hdWGCNA Analysis

**FIGURE 2:**
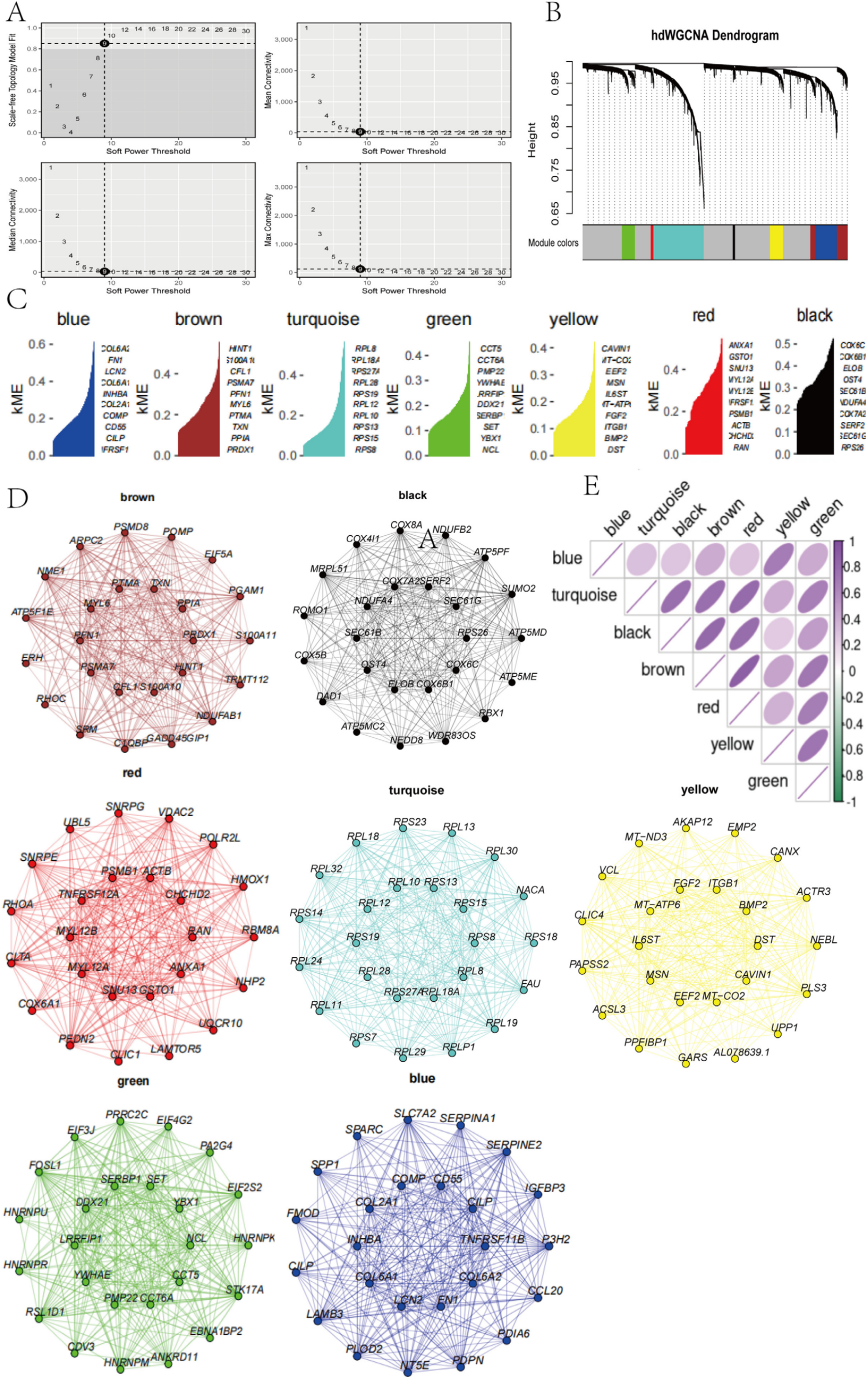
Characterising HTC‐specific Hub Genes analysis. (A, B) Selection of HTC subpopulations for hdWGCNA analysis at the single‐cell level. (C) Visualisation of differential genes in key modules, with higher KME indicating more significant differences. (D) PPI (protein–protein interaction) network visualisation of the seven modules. (E) Analysis of correlations among the key seven modules (yellow, brown, blue, turquoise, red, black, and green).

To further analyse the pathogenic genes in the HTC subpopulation, we selected HTC‐specific hub genes for analysis. Using the hdWGCNA method for clustering, we optimised the soft threshold coefficient to 9, minimising errors. This approach identified seven key modules: yellow, brown, blue, turquoise, red, black and green (Figure 2A–C). Interestingly, each module strongly correlated with OA progression (Figure 2E), and high‐expression gene regulatory networks for each module were visualised (Figure 2D).

### 3.3 Investigating the Impact of Hub Genes and Molecular Modules on OA Progression

**FIGURE 3:**
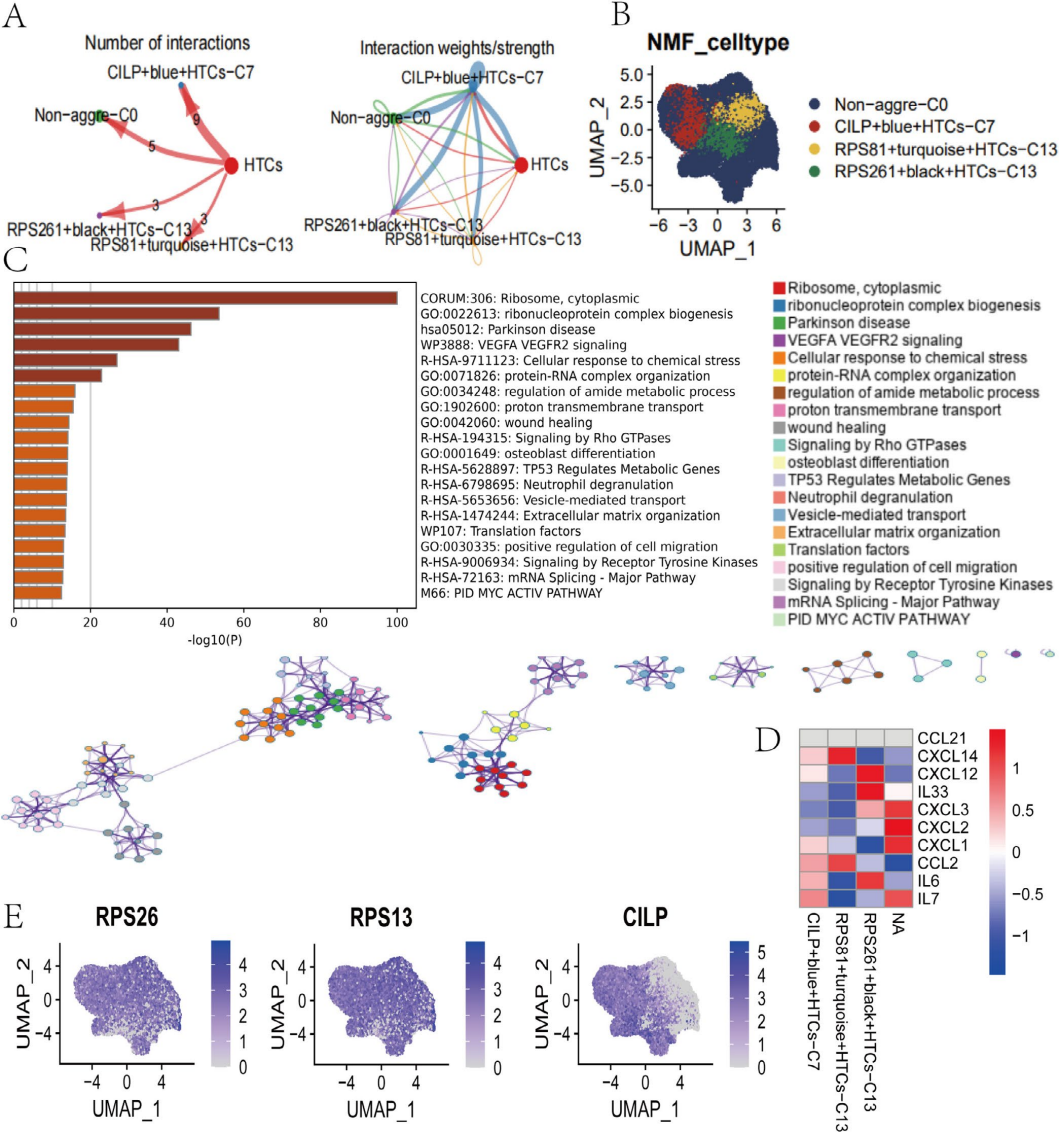
Exploring the role of CILP complexity in OA. (A) NMF selects key hub genes in the HTC group. (B) Clustering visualisation of NMF group genes. (C) KEGG enrichment analysis of hub genes. (D) Correlation analysis of RPS26, RPS13 and CILP genes with inflammatory factors. (E) Expression of RPS26, RPS13 and CILP genes.

We analysed the top five hub genes from each module to explore the key factors driving OA progression. NMF analysis revealed enrichment of CILP + blue, RPS261 + black, and RPS81 + turquoise, with CILP + blue showing the highest enrichment (Figure 3A,E). These modular genes exhibited strong cell communication links in the HTC group and were highly significant in the extracted HTC subgroup (Figure 3B). KEGG enrichment analysis revealed significant enrichment in the VEGFA signaling pathway (Figure 3C). Additionally, CILP showed a strong association with inflammatory factors (Figure 3D), suggesting that CILP may contribute to chronic inflammation and vascular remodeling during OA progression.

### 3.4 Exploring the Impact of CILP on Ferroptosis‐Related Genes in OA Progression

**FIGURE 4:**
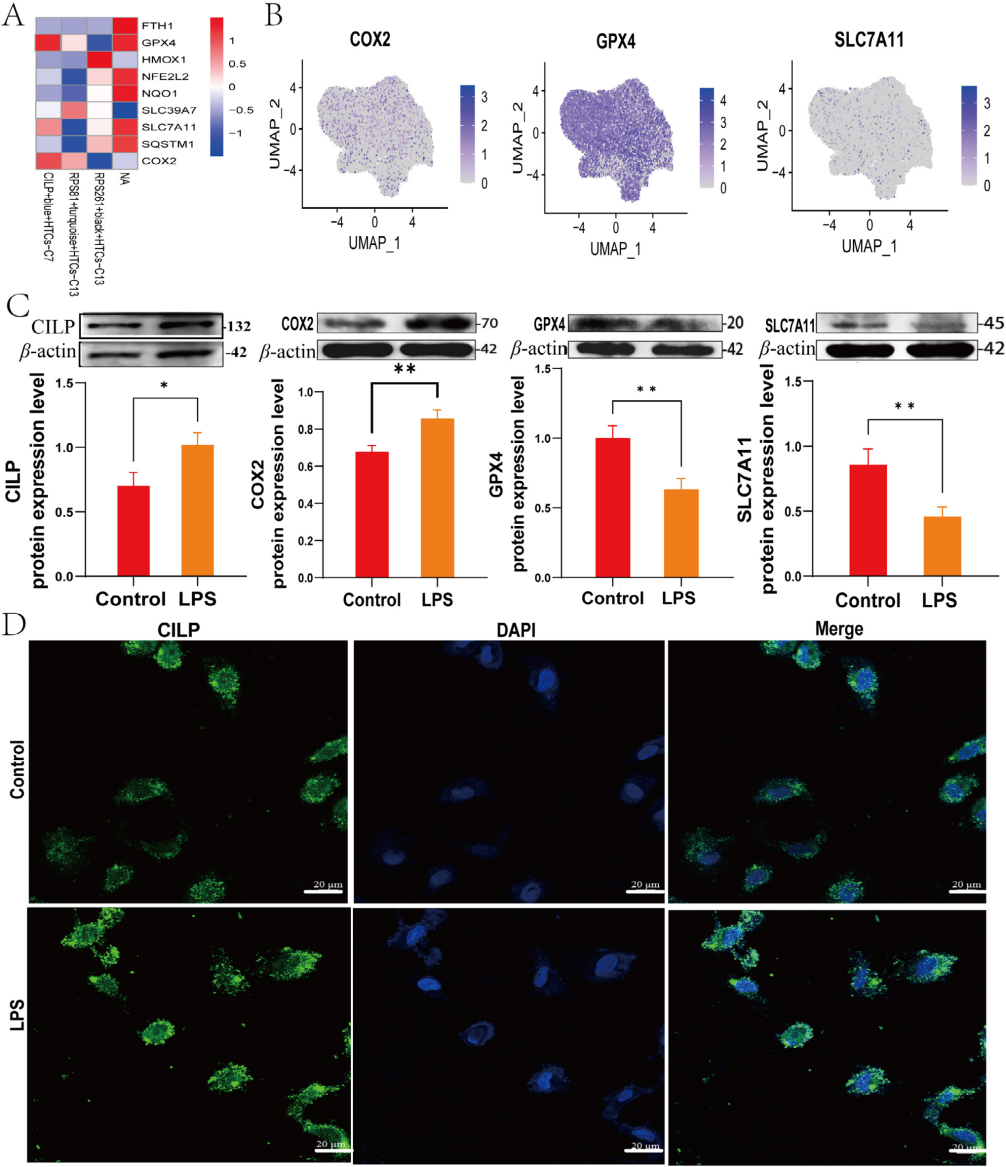
CILP‐mediated regulation of ferroptosis‐related genes in OA development. (A) Enrichment analysis of key ferroptosis genes. (B) Localization and expression of ferroptosis‐related genes. (C) The protein expression levels of CILP, COX2, SLC7A11 and GPX4 were detected by Western blot (*n* = 3). (D) Immunofluorescence staining for CILP (green) and DAPI (blue) under LPS induction. Scale bar, 20 μm. (*p < 0.05 and **p < 0.01).

Ferroptosis has been implicated in chronic inflammation regulation, and its role is closely linked to OA progression [30]. We performed a ferroptosis enrichment analysis on the CILP + blue, RPS261 + black, and RPS81 + turquoise genes and found that CILP promotes the expression of COX2, SLC7A11 and GPX4 (Figure 4A), which were enriched in HTCs (Figure 4B). Cellular‐level verification of CILP, COX2, SLC7A11 and GPX4 expression showed that LPS induction elevated the levels of CILP, COX2 and SLC7A11, while significantly decreasing GPX4 expression (Figure 4C). Immunofluorescence analysis further confirmed that CILP expression was elevated under LPS induction (Figure 4D). These findings suggest that CILP is critical in regulating ferroptosis‐related genes during OA progression.

### 3.5 miR‐140‐3p Targeting CILP Inhibits Ferroptosis Progression

**FIGURE 5:**
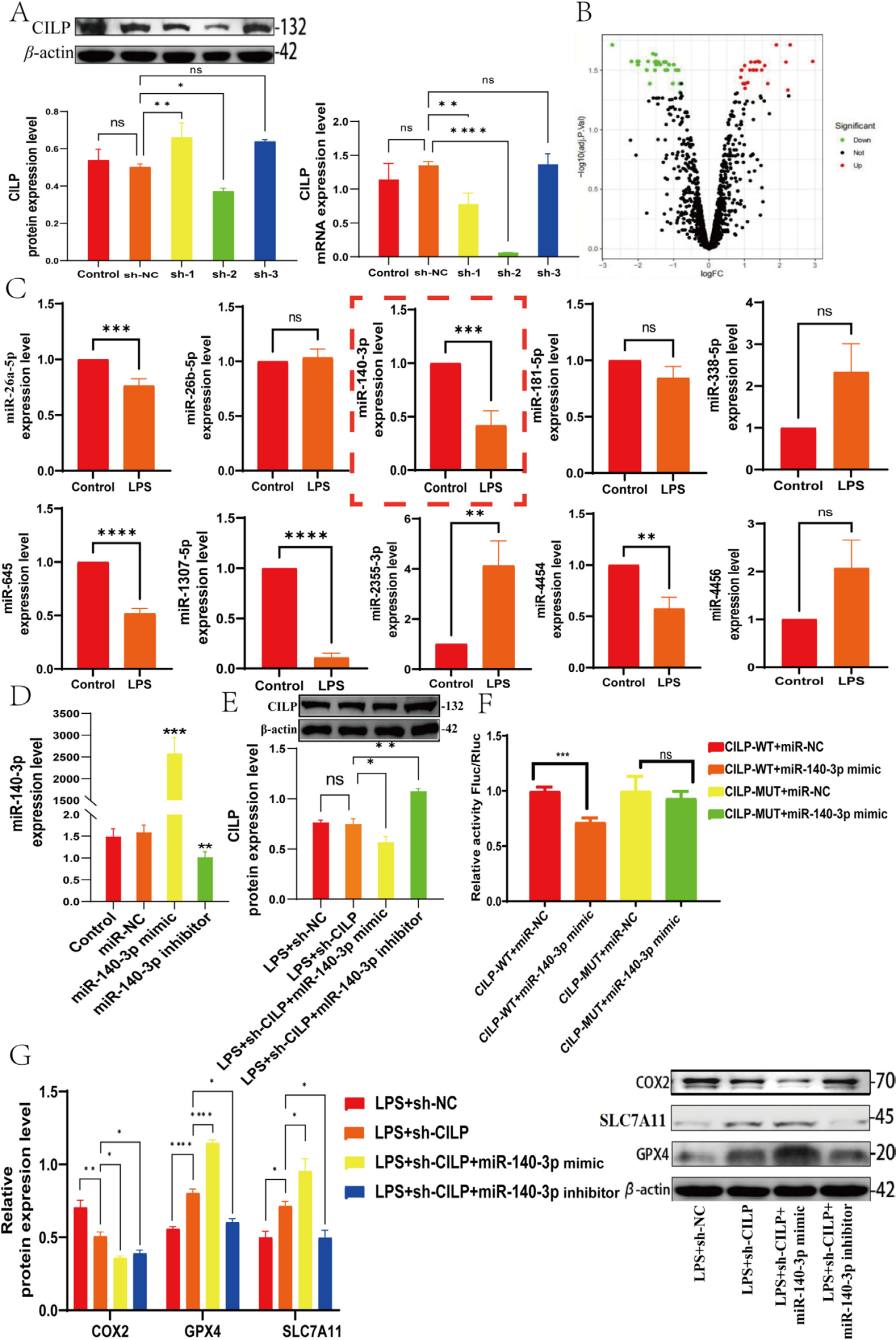
Upregulation of miR‐140‐3p inhibits the progression of ferroptosis. (A) Detection of CILP protein and mRNA expression after transfection with sh‐CILP‐1/‐2/‐3 (*n* = 3). (B) Volcano plot displaying differentially expressed miRNAs, with downregulated genes shown in green and upregulated genes shown in red. (C) qRT‐PCR was used to detect the expression of top 10 differentially expressed miRNAs (*n* = 3). (D) Expression status of miR‐140‐3p after overexpression and interference (*n* = 3). (E) The protein expression of CILP was detected by Western blot (*n* = 3). (F) Dual‐luciferase reporter assay to validate the binding of miR‐140‐3p with CILP (*n* = 3). (G) Expression levels of COX2, SLC7A11 and GPX4 (*n* = 3). (*p < 0.05, *p < 0.01, **p < 0.001, ***p < 0.0001).

Molecular targeted regulation is critical in understanding the regulatory mechanisms of OA. To identify key miRNAs involved in the progression of OA, we analysed the GEO dataset GSE79258 and found 25 downregulated and 30 upregulated miRNAs. The top 10 differentially expressed miRNAs were selected for further analysis (Figure 5B), and their effects on CILP gene expression were investigated (Figure 5A). Among these miRNAs, miR‐140‐3p, miR‐26a‐5p, miR‐645, miR‐4454 and miR‐1307‐5p showed decreased expression. No significant changes were observed for miR‐26b‐5p, miR‐181‐5p, miR‐338‐5p and miR‐4456, while miR‐2355‐3p showed increased expression (Figure 5C). Interestingly, among these six differentially expressed miRNAs, only miR‐140‐3p was found to directly target CILP, as confirmed by dual‐luciferase reporter assays (Figure 5F). To further explore the regulatory role of miR‐140‐3p on CILP, C28/I2 cells were transfected with either miR‐140‐3p mimic or inhibitor (Figure 5D). Overexpression of miR‐140‐3p promoted CILP expression, whereas inhibition of miR‐140‐3p resulted in a reduction of CILP (Figure 5E). Building on previous research, we confirmed that CILP is a downstream factor in regulating both inflammatory responses and ferroptosis. To further investigate the impact of miR‐140‐3p on ferroptosis regulation, we co‐transfected sh‐CILP with miR‐140‐3p mimic and inhibitor under LPS induction. Notably, in the LPS + sh‐CILP + miR‐140‐3p mimic group, there was an increase in the SLC7A11 and GPX4, while COX2 expression decreased. In contrast, in the LPS + sh‐CILP + miR‐140‐3p inhibitor group, SLC7A11 and GPX4 levels were reduced, but COX2 expression was elevated (Figure 5G). Collectively, these data suggested that miR‐140‐3p regulates ferroptosis‐related gene expression by directly targeting CILP.

### 3.6 The Regulatory Role of miR‐140‐3p in Controlling Ferroptosis and Inflammatory Factors in OA Development

**FIGURE 6:**
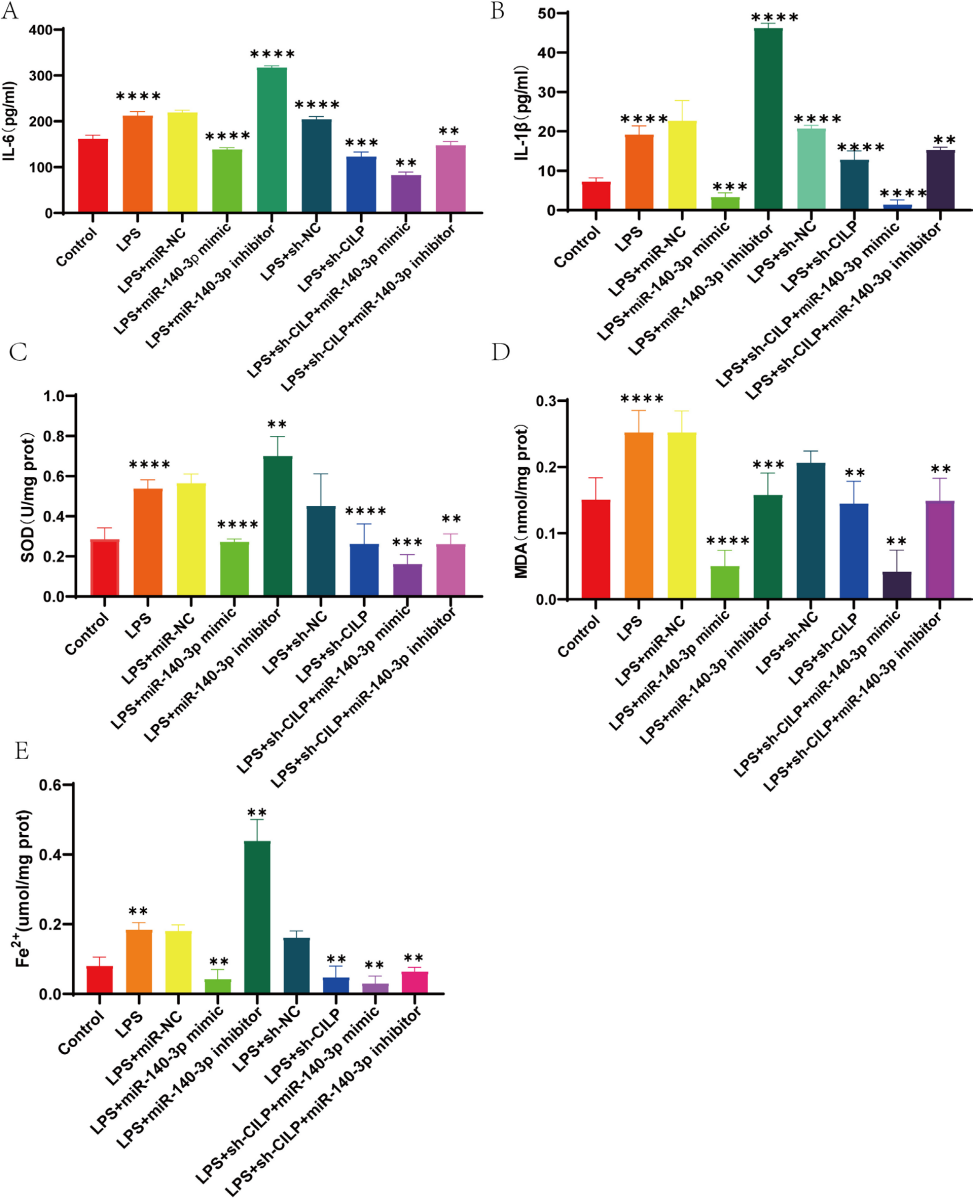
The regulation of miR‐140‐3p on ferroptosis and inflammatory factors in the development of OA. The IL‐6 contents (A); IL‐1β contents (B); SOD expression levels (C); MDA expression levels (D); Fe^2+^ expression levels (E) were analysed by ELISA after the cells were transfected with sh‐CILP, miR‐140‐3p mimic and inhibitor (*n* = 3). (*p < 0.01, **p < 0.001, ***p < 0.0001).

Building on the previous findings that miR‐140‐3p inhibits ferroptosis by targeting CILP, we explored whether miR‐140‐3p affects OA progression through other pathways, particularly in inflammation and OS. These factors are crucial in the pathogenesis of OA and are interconnected with ferroptosis. We first examined the levels of inflammatory markers IL‐6 and IL‐1β in cells. Our results showed that interference with CILP led to a reduction in IL‐6 and IL‐1β expression. However, transfection with the miR‐140‐3p inhibitor reversed this inhibitory effect (Figure 6A,B). Additionally, we measured SOD, MDA, and Fe^2+^ levels in cells co‐transfected with sh‐CILP, miR‐140‐3p mimic and inhibitor under LPS induction. Interfering with CILP reduced the levels of SOD, MDA, and Fe^2+^, and overexpression of miR‐140‐3p also inhibited these markers of OS. Conversely, the miR‐140‐3p inhibitor reversed the effects of CILP interference (Figure 6C–E). In conclusion, miR‐140‐3p is a critical regulator in OA progression by targeting CILP and regulating inflammatory and OS pathways, as well as ferroptosis, which collectively contribute to OA pathogenesis.

## 4 Discussion

OA, a common joint disorder affecting millions of individuals globally, is marked by the progressive degradation of articular cartilage and abnormal bone growth, severely limiting mobility, independence, and quality of life, particularly as the population ages [31, 32]. While current treatments primarily aim to manage pain, reduce inflammation, and improve physical function through medication and exercise [33, 34], they largely focus on symptom management rather than halting or reversing the progression of the disease [35]. This limitation underscores the urgent need for new therapeutic strategies that can modify the course of OA. In this study, we have identified miR‐140‐3p as a key molecular regulator in the progression of OA. miR‐140‐3p significantly influences the biological functions of chondrocytes by modulating CILP expression, a protein vital for maintaining and repairing joint tissues. Our findings demonstrated that miR‐140‐3p downregulates CILP expression, highlighting its potential as a therapeutic target in OA. Furthermore, we discovered that miR‐140‐3p has another promising characteristic: its ability to inhibit ferroptosis, a form of regulated cell death that has been implicated in the pathogenesis of OA. By targeting these pathways, miR‐140‐3p shows potential as a novel therapeutic candidate capable of not only alleviating symptoms but also addressing the underlying disease mechanisms.

Molecular signatures correlated with synovitis in OA were identified by examining hub genes in OA samples compared with normal controls. KEGG pathway enrichment revealed that CILP, a key regulatory molecule, is linked to inflammation and ferroptosis. Ferroptosis, a form of programmed cell death triggered by disruptions in iron homeostasis, plays a significant role in various inflammatory and metabolic disorders [36, 37, 38]. These findings suggest that CILP may contribute to the progression of OA by regulating both inflammation and ferroptosis in chondrocytes. The function of CILP as a hub gene will be further validated through a series of cell experiments.

MiRNAs have been implicated in the pathogenesis of OA, with several miRNAs playing significant roles in the disease's progression [39, 40]. Understanding the roles of miRNAs and their mechanisms in OA is essential for identifying potential biomarkers or therapeutic targets [41]. This study used data from the GEO database for bioinformatics analysis to identify differentially expressed miRNAs, with qRT‐PCR further validating the expression. Six differentially expressed miRNAs (miR‐140‐3p, miR‐26a‐5p, miR‐645, miR‐4454, miR‐1337‐5p and miR‐2355‐3p) were screened, and a dual luciferase assay confirmed that only miR‐140‐3p targets CILP. Building on these findings, we further explored the interaction between miR‐140‐3p and key signaling molecules involved in inflammatory and ferroptosis pathways. Previous studies have shown that miR‐140‐3p can modulate the JAK2/STAT3 signaling by targeting HMGB1, reducing airway inflammation and remodeling in asthma [42]. Zhou et al. [43] demonstrated that miR‐140‐3p targets DACH1 to suppress HMC growth and release inflammatory cytokines. Chen et al. [44] reported that dexmedetomidine increased miR‐140‐3p levels in RLE‐6TN cells, inhibiting PD‐L1 expression and the JNK‐Bnip3 pathway, thereby alleviating inflammation and type II alveolar cell injury. These results highlight the role of miR‐140‐3p in various diseases by regulating inflammation. However, few studies have investigated its effect on ferroptosis.

The experiments demonstrated that upregulating miR‐140‐3p effectively targets CILP expression. According to the results of our study, miR‐140‐3p may play a role in the ferroptosis process of OA, though its precise function in ferroptosis during OA progression requires further investigation. Therefore, we further co‐transfected sh‐CILP with miR‐140‐3p mimic and inhibitor in chondrocytes under LPS induction. The results showed that miR‐140‐3p inhibited the expression of GPX4 and SLC7A11 proteins while promoting COX2 expression by targeting CILP. These findings highlight miR‐140‐3p's crucial involvement in the ferroptosis pathway, modulating key iron‐related proteins to regulate cellular iron balance and influence ferroptosis activation. Furthermore, transfection with the miR‐140‐3p inhibitor reversed the impact of CILP interference on inflammatory markers such as IL‐6, IL‐1β, SOD, MDA, and Fe^2+^ levels. These results enhance our understanding of miR‐140‐3p's involvement in inflammation, underscoring its potential as a therapeutic target for inflammation‐driven conditions. This study also reveals the complexity of miR‐140‐3p's regulation of ferroptosis and inflammatory processes, suggesting that miR‐140‐3p‐based therapies could offer novel approaches to managing inflammatory and metabolic diseases. Future research should further clarify the mechanisms by which miR‐140‐3p operates in various pathological contexts, with the goal of developing targeted treatments for these conditions.

However, this study has several limitations. First, a small sample size was used to screen for hub genes and miRNAs. Future studies with larger sample sizes or the integration of multiple datasets would help improve the validity of the findings. Second, while cellular functional experiments were conducted, animal studies and clinical investigations were lacking. Comprehensive validation through these approaches would significantly enhance the diagnostic and therapeutic value of the OA biomarkers identified here. Finally, a more thorough understanding of the hub‐gene targets in OA is essential for identifying effective therapeutic strategies.

## 5 Conclusion

In summary, we demonstrated for the first time that miR‐140‐3p is dysregulated in OA samples and chondrocytes under LPS induction. Mechanistically, miR‐140‐3p regulates ferroptosis, inflammation, and OS in chondrocytes by targeting CILP. Our findings underscore the critical role of the miR‐140‐3p/CILP axis in OA pathogenesis, suggesting that targeting this axis could offer a promising strategy for treating OA. This study deepens our understanding of miR‐140‐3p's involvement in OA and provides a theoretical basis and experimental evidence for developing new therapeutic approaches. These efforts are crucial for identifying novel biomarkers and therapeutic targets, potentially transforming personalised medicine for OA.

## Author Contributions

F.M., L.W. and H.C. drafted the manuscript. F.M., L.W., H.C., X.L., Y.X., K.C., J.Z., R.Y., J.L., K.X. and X.Y. performed the literature search and collected the data. L.W. completed in vitro experiments. L.W. analysed and visualised the data. L.W. helped with the final revision of this manuscript. All authors reviewed and approved the final manuscript.

## Ethics Statement

The Ethics Committee of Ningxia Medical University waived the requirement for ethics approval and the need to obtain consent.

## Conflicts of Interest

The authors declare no conflicts of interest.

## Data Availability

The datasets analysed in this study are available in the GEO database (https://www.ncbi.nlm.nih.gov/geo/). Additionally, the datasets used in this study can be obtained from the corresponding authors upon reasonable request.
